# Against Me

**Published:** 1870-10

**Authors:** 


### AGAINST ME.

There are times when things go amiss, or without apparent cause a sense of depression comes over a man, and he feels as if all the world were against him ; that everybody is selfish, and that every man is a rascal, seeking to cheat him out of all he possesses or has left. A man came to me the other day with the bearing, and dress, and language of a gentleman of culture. The meeting was wholly accidental. He brought to my remembrance that five years before he came to my door in a carriage, leaving a near relative of mine. Then he had reached New York with forty thousand dollars of his own earnings, every dollar of it legitimately made. Said he, "I have walked the streets of New York for three days, eating but once for the want of means to eat oftener ; I sat upon the curbstone, ready to die ; a stranger spoke to me, and seeing my condition, said, 'I have no money, but you can come to my place a week, and that will give you time to look around.' This is Saturday, and to-night the week ends ; whether I can stay until Monday, I cannot tell." He was in the last stages of consumption, and had to rest every block or two. Said he, "I lost most of my money in legitimate business ; but trade took an adverse turn ; some was lost in loans to friends whom I wanted to help along—a hundred dollars here—five hundred there—a thousand yonder ; none of it ever came back to me. Friends, in turn, have lent me money ; I cannot ask them for more ; my last resource is the Hospital, and there I must go."

I felt as if I wanted to see the man who could thus invite a stranger to his "place," and sent a message to him to come and see me. I afterwards learned that this good Samaritan was a Baptist preacher.

Reader, when you get low-spirited again, just think of this incident, and be assured that, scattered over the wide world, there are other hearts just as noble, only needing the opportunity to show that nobility.

Another noble man maintains a house at his own expense in Pearl Street, New York City, where any respectable person in utter destitution can find shelter and a bed, without cost for a short time. But who supplies the money, no man can find out. Who shall not say, "There is a mansion in Heaven for him, any how;" and as long as there are such in the world, don't say again, "Everybody is a rascal."

